# Improving reusability along the data life cycle: a regulatory circuits case study

**DOI:** 10.1186/s13326-022-00266-4

**Published:** 2022-03-28

**Authors:** Marine Louarn, Fabrice Chatonnet, Xavier Garnier, Thierry Fest, Anne Siegel, Catherine Faron, Olivier Dameron

**Affiliations:** 1 Univ Rennes, CNRS, Inria, IRISA, UMR 6074, Rennes, F-35000 France; 2 UMR_S1236, Université Rennes 1, INSERM, Etablissement Français du Sang, Rennes, 35000 France; 3 Laboratoire d’Hématologie, Pôle de Biologie, Centre Hospitalier Universitaire de Rennes, Rennes, 35033 France; 4 Université Côte d’Azur, Inria, CNRS, I3S, Sophia-Antipolis, France

**Keywords:** Dataset architecture, Bioinformatics, RDF named graphs, SPARQL, Reusability, Linked Open Data

## Abstract

**Background:**

In life sciences, there has been a long-standing effort of standardization and integration of reference datasets and databases. Despite these efforts, many studies data are provided using specific and non-standard formats. This hampers the capacity to reuse the studies data in other pipelines, the capacity to reuse the pipelines results in other studies, and the capacity to enrich the data with additional information. The *Regulatory Circuits* project is one of the largest efforts for integrating human cell genomics data to predict tissue-specific transcription factor-genes interaction networks. In spite of its success, it exhibits the usual shortcomings limiting its update, its reuse (as a whole or partially), and its extension with new data samples. To address these limitations, the resource has previously been integrated in an RDF triplestore so that TF-gene interaction networks could be generated with two SPARQL queries. However, this triplestore did not store the computed networks and did not integrate metadata about tissues and samples, therefore limiting the reuse of this dataset. In particular, it does not enable to reuse only a portion of *Regulatory Circuits* if a study focuses on a subset of the tissues, nor to combine the samples described in the datasets with samples from other studies. Overall, these limitations advocate for the design of a complete, flexible and reusable representation of the *Regulatory Circuits* dataset based on Semantic Web technologies.

**Results:**

We provide a modular RDF representation of the Regulatory Circuits, called *Linked Extended Regulatory Circuits* (LERC). It consists in (i) descriptions of biological and experimental context mapped to the references databases, (ii) annotations about TF-gene interactions at the sample level for 808 samples, (iii) annotations about TF-gene interactions at the tissue level for 394 tissues, (iv) metadata connecting the knowledge graphs cited above. LERC is based on a modular organisation into 1,205 RDF named graphs for representing the biological data, the sample-specific and the tissue-specific networks, and the corresponding metadata. In total it contains 3,910,794,050 triples and is available as a SPARQL endpoint.

**Conclusion:**

The flexible and modular architecture of LERC supports biologically-relevant SPARQL queries. It allows an easy and fast querying of the resources related to the initial *Regulatory Circuits* datasets and facilitates its reuse in other studies.

**Associated website:**

https://regulatorycircuits-lod.genouest.org

## Background

**Limits to data reusability in life sciences** In life science, there has been a long-standing effort to standardize and integrate reference datasets and databases [1, 2]. Despite these efforts, many studies data are provided using specific and non-standard formats [3]. This hampers the capacity to reuse the studies data in other pipelines, the capacity to reuse the pipelines results in other studies, and the capacity to enrich the data with additional information [4].

**The Regulatory Circuits project** The *Regulatory Circuits* project [5, 6] is one of the largest efforts for integrating human cell genomics data. It has been cited 217 times (Google Scholar – 17 May 2021). Its data originate from several independent programs such as FANTOM5 [7] and ENCODE [8], and are aggregated by a complex in silico pipeline. A major output of these analyses, used in at least 42 other articles, consists in 394 tissue-specific networks describing the interactions between transcription factors (TFs) and their target genes in each tissue. From a biological perspective, the regulation of a gene by a TF results from two mechanisms, as TFs can bind to two types of regulatory regions called promoters and enhancers. The 394 tissue-specific networks are represented by weighted oriented graphs in which TFs are connected to genes. They are provided as 394 tabulated files, which differ only by the values of the weights associated with the interactions (a null value meaning that the considered interaction is not predicted in the considered tissue). Therefore, the pipeline complexity lies in the computation of the scores, which is based on two main features: (i) the combination of the respective contributions of the enhancer and promoter regions to the predicted strength of the TF-gene regulation and (ii) the aggregation of the information on the different samples (from 1 to 33) which constitute each tissue (808 cell-samples analyzed in total).

The main drawback with the *Regulatory Circuits* original resource is the lack of intermediary results: only the input files (some with pre-processing) and the resulting tissue networks are accessible. The resource does not describe the specific contributions from the different samples nor the specific contributions from the enhancers and the promoters in the tissue networks. Another drawback is the lack of reproducibility of the pipeline that leads to the impossibility to compute those intermediary networks. As the regulation scores in each tissue-specific network depend on the weights of the enhancers and the promoters in all the samples, these two drawbacks make it impossible (i) to update *Regulatory Circuits* along the successive releases of the resources it is based on, (ii) to reuse only a portion of *Regulatory Circuits* if a study focuses on a subset of the tissues, and (iii) to combine the *Regulatory Circuits* samples with samples from other studies.

**Semantic Web technologies** Semantic Web technologies have long been perceived as a relevant framework for supporting data integration and reuse [9], and have been widely adopted [10, 11], but some challenges remain to achieve Web-scale integration [12]. This is of particular importance as life sciences are on par with other Big Data domains in terms of data quantity, are probably facing a higher complexity of data, and this trend is expected to get worse over the next decade [13].

**RDF data structure for the Regulatory Circuits project.** In a previous study [14], we provided an RDF data repository containing the input of the *Regulatory Circuits* resource, consisting of 3,226,341 entities and 335,429,988 relations between them. As an application case-study, we demonstrated that TF-gene interaction networks through promoter and enhancer for each cell sample could be generated on-the-fly with two SPARQL queries. Those two queries are time efficient to retrieve the TF-genes relations of enhancer sample-specific networks and promoter sample-specific. However, those queries do not combine the scores from enhancer and from promoter nor combine the samples constituting a tissue to obtain its regulatory network. The solution proposed does not store the computed networks and requires to be computed each time a user may need them. Another limitation in the use of this resource is the lack of metadata. These are necessary to identify the subset of the samples that meet a user’s specific criteria when reusing *Regulatory Circuits*. Overall, these limitations require a more complete, more flexible and reusable representation of the *Regulatory Circuits* dataset.

**Expected benefits of RDF technologies** In this article, we elaborate upon the strategy of [14] to generate a public RDF resource which contains not only the *Regulatory Circuits* source biological data (already integrated in [14]), linked to standard Linked Open Data resources, but also the results of the analysis pipeline at the sample and tissue-specific layers. The expected benefits are threefold. First, instead of only having access to tissue-specific regulatory networks, it will also be possible to query this resource from different perspectives. For example, one may be interested in comparing the targets of a given TF in different tissue-specific networks, or in determining how the TFs regulating a given gene vary among networks. Second, new tissue-specific regulatory networks may be defined based on the 808 samples from *Regulatory Circuits*. This encompasses both specializing a network by selecting a subset of the samples it is based on, or generalizing a network by adding other samples. Third, it will allow to combine the data from *Regulatory Circuits* with user-specific samples, to extend the resource.

**Approach** First, we designed the structure of the sample-specific graphs as well as the SPARQL queries for computing the weights associated to TF-genes relations. Second, we designed the structure of the tissue-specific graphs as well as the SPARQL queries for computing the weights and scores associated to TF-genes relations based on the values computed for the samples. Third, we described the biological data associated to the samples and the tissues, we complemented them with mappings to external public databases, and used them to enrich the original dataset with an experimental context graph. Finally, we proposed a modular organization of the aggregated datasets into RDF named graphs linked by an additional metadata graph, which allows to identify the relevant portions of the dataset in order to maintain query performances.

**Linked Extended Regulatory Circuits (LERC), a flexible resource to query tissue- and sample-specific TF-genes interactions**. We provide an RDF representation of the *Regulatory Circuits*, called *Linked Extended Regulatory Circuits (LERC)*. It consists in (i) descriptions of biological and experimental context, linked to the references databases, (ii) annotations about TF-gene interactions at the sample level for 808 samples, (iii) annotations about TF-gene interactions at the tissue level for 394 tissues, (iv) metadata connecting the three above listed named graphs. Overall, the resource contains 3,910,794,050 triples and is available as a Virtuoso endpoint at https://regulatorycircuits-lod.genouest.org/ [15]. We show how our flexible architecture supports biologically-relevant SPARQL queries that were not possible with our previous representation of *Regulatory Circuits*’s final results in RDF.

This integration scheme used to construct *LERC* is applicable to any similar dataset produced in other projects.

## Methods

All the results presented in the paper were obtained by relying on Semantic Web technologies. Our strategy was to create an RDF graph for each different dataset handled in the regulatory circuits project (biological dataset, experimental context dataset, 394 tissue-specific TF-gene datasets, 808 sample-specific TF-gene datasets). Then, the capability of the RDF language to identify groups of related triples as *named graphs* was used to link all the RDF datasets together. This modular design allows (1) to assign metadata describing each group, (2) to improve SPARQL queries performances by only considering some relevant portions of a dataset, (3) to extend the dataset by adding new groups such as new samples data and (4) to reuse some portions of the dataset in other studies. Note that this addresses the limits to data reusability identified at the beginning of the Background section.

### Biological data from *Regulatory circuits*

The *Regulatory Circuits* website and supplementary data give access to unstructured, disconnected and diversely formatted tabulated files related either to input biological data (FANTOM5 data, genes and regions genomic coordinates, TFs binding sites occurrences...) or computation intermediate results (59 files). The main output of the in silico analyses resulting from the *Regulatory Circuits* project consists in maps (called networks) describing interactions between TFs and their target genes in each of the 394 studied tissues.

Each network is described by an oriented graph in which TFs are connected to genes. The nodes are annotated with biological information (gene IDs for both TFs and target genes). The edges are annotated with a unique score aggregating two different weights representing the respective contributions of the enhancer and promoter regions to the predicted strength of the TF-gene regulation. These respective contributions as well as the formula used to compute the final score are neither described nor available. These 394 tissue-specific TF-gene interaction networks are provided as tabulated files, and the pipeline to produce them is neither usable nor reproducible.

### Biological data RDF graph

**Fig. 1:**
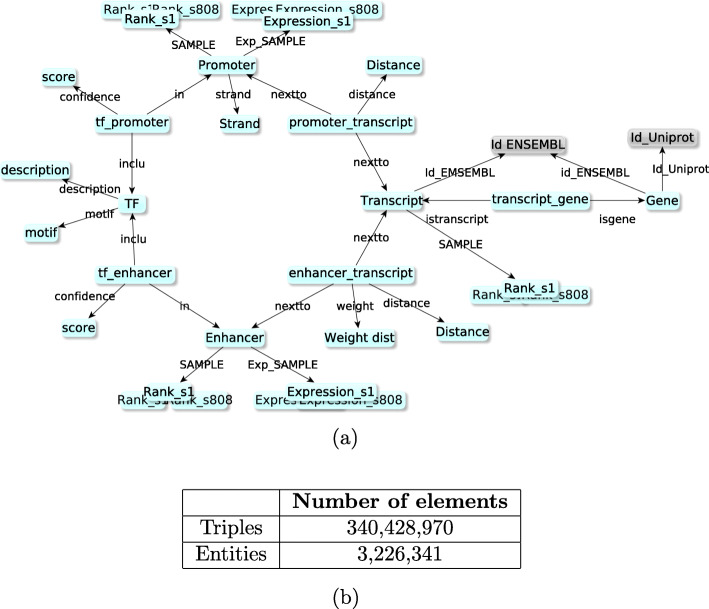
**a** Graphical representation of the structure of the biological data graph from the *Regulatory Circuits* project. Boxes represent classes of entities. The grey boxes represent mappings to external resources. **b** Data integrated into the biological data graph before running the injection queries. Biological data RDF graph structure of the RDF graph and its population

The biological data graph contains the minimal set of biological entities required to build the *Regulatory Circuits* networks together with their attributes (values), and describes the relations between these entities. As detailed in [14] and depicted in Fig. 1, it is based on five main types of biological entities: three related to genes or proteins (gene, transcript, TF) and two related to chromosomal regulation regions (promoter, enhancer), connected by five reified relations (see below).

The identifiers of genes (19,125 instances of the class *Gene*), transcripts (53,549 instances of the class *Transcript*) and transcription factors (691 instances of the class *TF*) are constructed based on the names provided by the *Regulatory Circuits* datasets (HGNC reference identifiers for *TF* and *Gene*, Ensembl transcript names for *Transcript*). These identifiers are linked to identifiers from external databases such as UniProtKB [16] (release 2021_04) and Ensembl [17] (release 104) as follows. Genes are associated to the UniProt identifier of their reviewed proteins; in case of several proteins being reviewed for a gene, the longest one is selected. Both genes and transcripts are associated to their Ensembl identifiers as already available in *Regulatory Circuits* datasets.

There are two classes of regulatory regions: *Promoter* (184,828 entities) and *Enhancer* (43,011 entities).

The dataset comprises five types of reified relations: two between TFs and regulatory regions weighed by the *confidence* of transcription factor binding site in the region (1,169,797 entities for *TF*_*promoter* and 524,816 for *TF*_*enhancer*), two between regulatory regions and transcripts weighed by the *distance* and the *Weight*_*Distance* between those entities (123,441 entities for *promoter*_*transcipt* and 950,514 for *enhancer*_*transcipt*), and a last one between transcripts and genes (53,449 entities). Each instance of classes *Promoter* or *Enhancer* is associated with two sets of 808 float values, one corresponding to its expression value in each sample, and the other corresponding to its normalized relative rank in each sample compared to the 807 others. Similarly, each instance of the *Transcript* class is associated with 808 float values, describing its normalized relative rank in each sample compared to the others. This rank information is directly provided by *Regulatory Circuits*. Contrary to the promoters and enhancers, no measured expression value is provided for transcripts. For the *LERC* resource, the ranks were computed according to the methodology described in [6], using the max of the transcript promoters’ rank. Each rank identifier is built by using the sample’s identifier (*libId*).

Figure 1 compiles the total number of triples and entities in the biological data graph.

### Sample-specific weights of the TF-gene regulation networks

Each TF-gene interaction is characterized by a promoter weight and by an enhancer weight. As shown in Fig. 1, the relation between a TF and a regulatory region is described by a *confidence* value, and the *rank* of the regulatory region is described by a value associated with the sample. The promoter weight is defined by $weightP=\max ((confidence \times \sqrt (Rank\_promoter\_sample*Rank\_transcript\_sample))^{2}) $, where the maximum is computed for all the possible promoters mediating the interaction. The enhancer weight is defined by $weightE=\max ((confidence \times Weight\_Distance \times \sqrt (Rank\_transcript\_sample \times Rank\_enhancer\_sample))^{2})$. These formulas were generated according to the method section of [6].

**Fig. 2:**
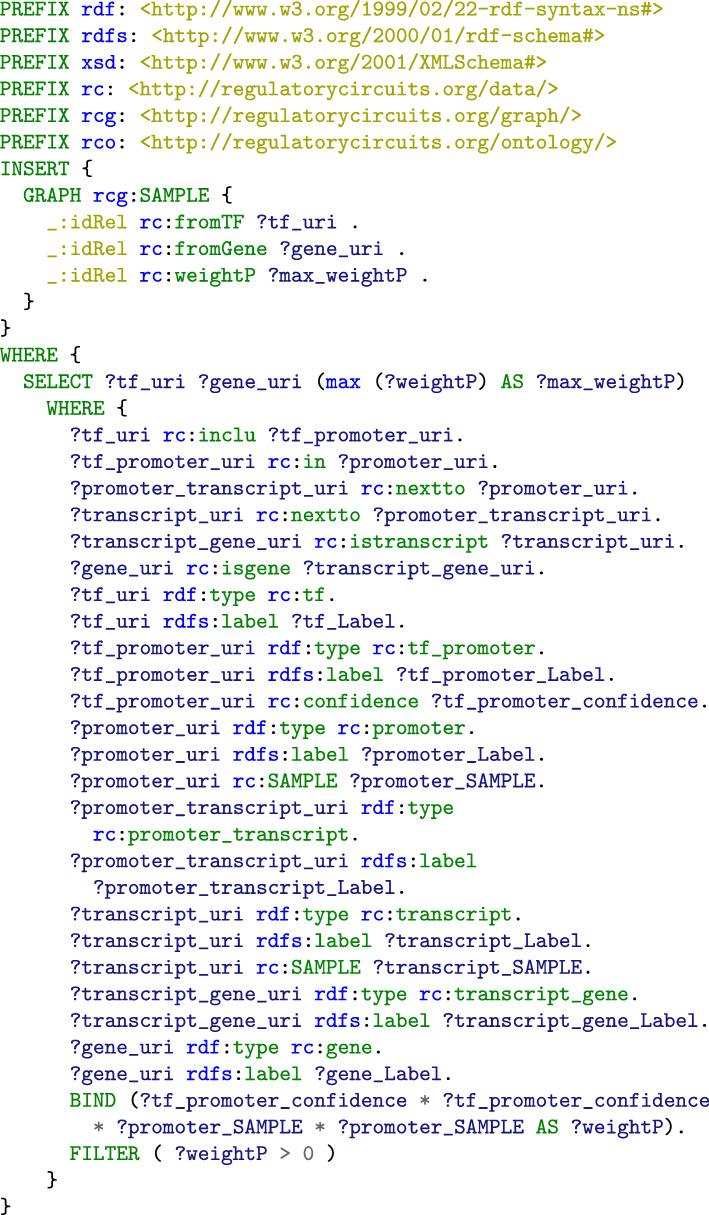
SPARQL query for computing the sample-specific value of the weight associated to promoters for the TF-gene regulation relations (in the WHERE clause) and inserting it in the corresponding sample graph (in the INSERT clause)

The SPARQL query for computing *weightP* is given in Fig. 2, where *SAMPLE* must be replaced by the identifier of an actual sample. A similar query for computing *weightE* is available on the GitHub repository of the project (cf. Availability section). The relations with a null weight are excluded to avoid overloading the graph.

Each sample-specific network contains some values such as ranks that depend on values from the other networks, so that the 808 sample-specific networks have to be computed simultaneously. In order to save time and CPU usage, we executed these queries once (11.2 days CPU times) and integrated the final 808 sample-specific networks in our resource triplestore, by using an INSERT operation as shown in Fig. 2.

### Tissue-specific weights of the TF-gene regulation networks

**Fig. 3:**
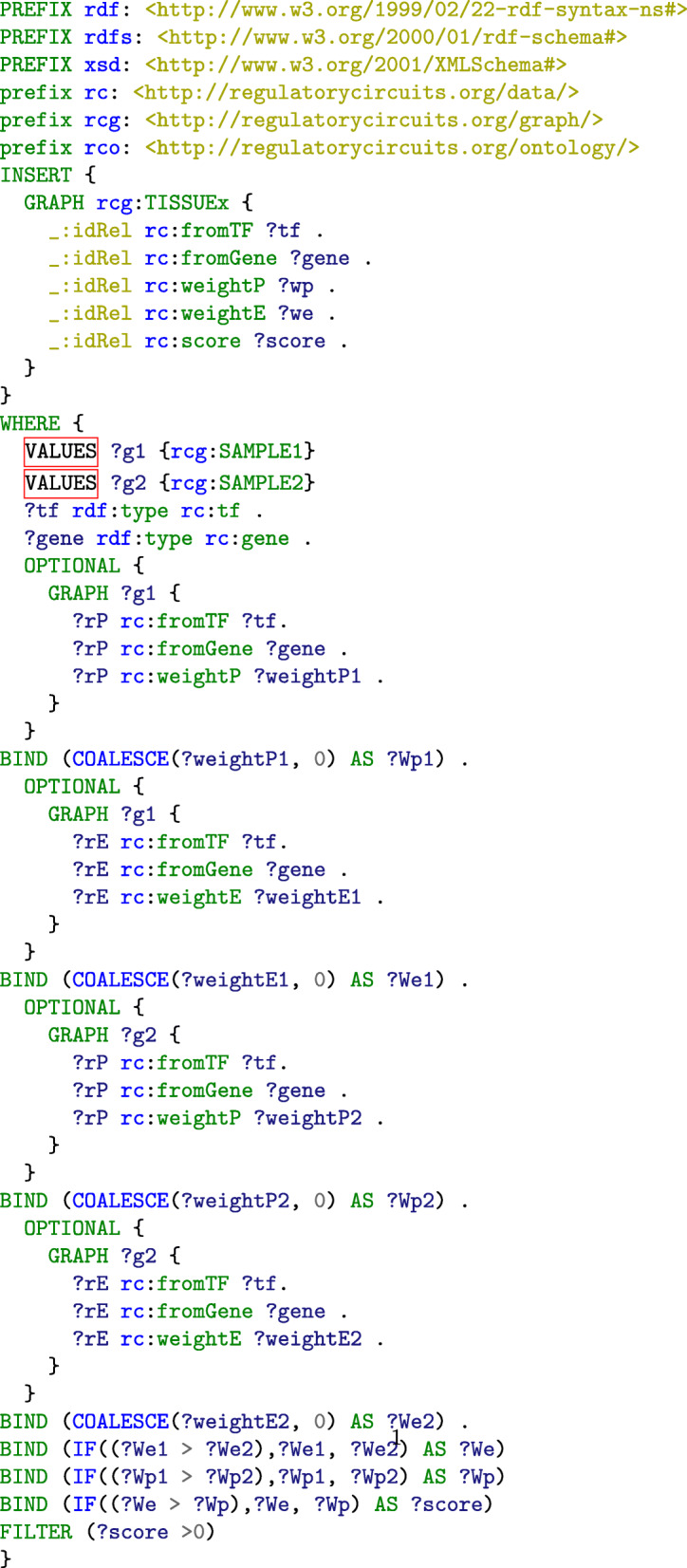
SPARQL query for computing tissue-specific values of the weights associated to promoters and enhancers and the global score for the TF-gene regulation relations from the values of the samples SAMPLE1 and SAMPLE2 associated to the tissue TISSUEx, and inserting them in the graph describing the corresponding tissue

At the tissue level, each TF-gene interaction is characterized by (i) a promoter weight (max of the promoter weights among the samples composing the tissue), (ii) an enhancer weight (max of the enhancer weights among the samples composing the tissue), (iii) a *Max score* combining the two previous one, and (iv) a *RC score* extracted from the *Regulatory circuits* output data files. We designed a SPARQL query to compute tissue-specific promotor/enhancer weights and MAX scores and re-inject them into the tissue-specific RDF graphs (Fig. 3). It computes the weights of TF-Gene relations in a tissue-specific network formed by two separate samples. The queries for tissue-specific network with more samples (up to 33) or a single sample are available in the GitHub repository of the project.

### Experimental context graph

The *experimental context graph* describes the experimental information about the 808 samples (cell types, organs, patient clinical data [age, gender..], diseases...), about the 394 tissues (linked to the samples they are composed of) as well as the mappings to reference databases. Note that the experimental data (expressions and ranks) belong to the biological data graph. All the information contained in the experimental context graph are extracted from the nmeth_.3799-S2.xlsx file present in the *Regulatory Circuits* supplementary data, and formatted to respect the identifiers of the generated samples-specific or tissue-specific RDF graphs.

When applicable, we also include links to other knowledge bases from the Linked Open Data such as gene identifiers from Ensembl, protein identifiers from UniProtKB, cell types and anatomical structures from the Uberon and the Foundational Model of Anatomy ontologies.

### Metadata graph

The *metadata graph* contains all the information about the other graphs including their VoID descriptions, as well as the associations of the samples and tissues from the experimental context graph with their respective graph containing their specific regulatory network.

This explicit representation of the metadata about the samples and the tissues can be queried by the users for identifying the subset of samples or tissues they are interested in. The modular approach described next allows the user to retrieve the corresponding portions of the dataset.

### Structuration and computation of the modular graphs

We took advantage of the notion of named graph in the RDF model to design a modular structure for *Regulatory Circuits* that makes it possible to identify the subset of the samples and tissues that meets the user’s requirements, to retrieve the corresponding networks and to combine them with additional data. To do so, we first created an RDF named graph for general biological data such as the binding and neighborhood relations between TFs, regulatory regions and genes. Second, we created a distinct RDF named graph for each sample- and tissue-specific network (see the INSERT clauses of the queries in Figs. 2 and 3 that generate the weights and scores of regulation relations in specific graphs based on information from the biological data graph). Third, an additional metadata graph associates each of these named graph with the corresponding sample or tissue. Fourth, the samples and tissues’ descriptions (i.e. the organs, cell types as well as patient’s characteristics) as well as the composition relations of tissues into samples are represented in the experimental context graph. Thus, a user can query the experimental context graph to identify the samples and tissues that meet some constraints, and retrieve the associated networks. Likewise, new samples or tissues can be combined with *Regulatory Circuits* by adding the corresponding graphs and generating the associated metadata and experimental context graphs. In both cases, the weights and scores for the regulation relations of new dataset can be re-computed with the queries from Figs. 2 and 3, addressing the reusability and reproducibility requirements.

### Availability

The original datasets of the Regulatory Circuits project were downloaded as tabulated files from the website of the original project [5].

All data related to *Linked Extended Regulatory Circuits (LERC)* resource are available on the website of the project: https://regulatorycircuits-lod.genouest.org. The RDF version of the dataset is under the Attribution 4.0 International (CC BY 4.0) license. The SPARQL queries used to generate the sample and tissue-specific TF-gene graphs are available on GitHub https://github.com/mlouarn/RCsparql/. The generated turtle files are available at https://zenodo.org/record/4889146.

## Results

### Computation and integration of 808 weighted TF-gene interactions sample-specific graphs

According to *Regulatory Circuits* published methodology, the TF-gene interactions are mediated by the ability of the TF to bind into regulatory regions of the chromatin (enhancers or promoters), the distance of this region to the gene (enhancer being farther and promoter being adjacent to the genes), the region accessibility and the gene expression. Each TF-gene interaction is therefore characterized by a promoter weight and by an enhancer weight (see details in Methods). From the 808 samples, the weights were computed by adapting the method introduced in [14]. The method consisted in using the biological data graph to compute the weights with a SPARQL query and to use the same query - with an INSERT operation - to inject the result of the query in the data graph of the sample-specific TF-gene interaction network.

**Fig. 4:**
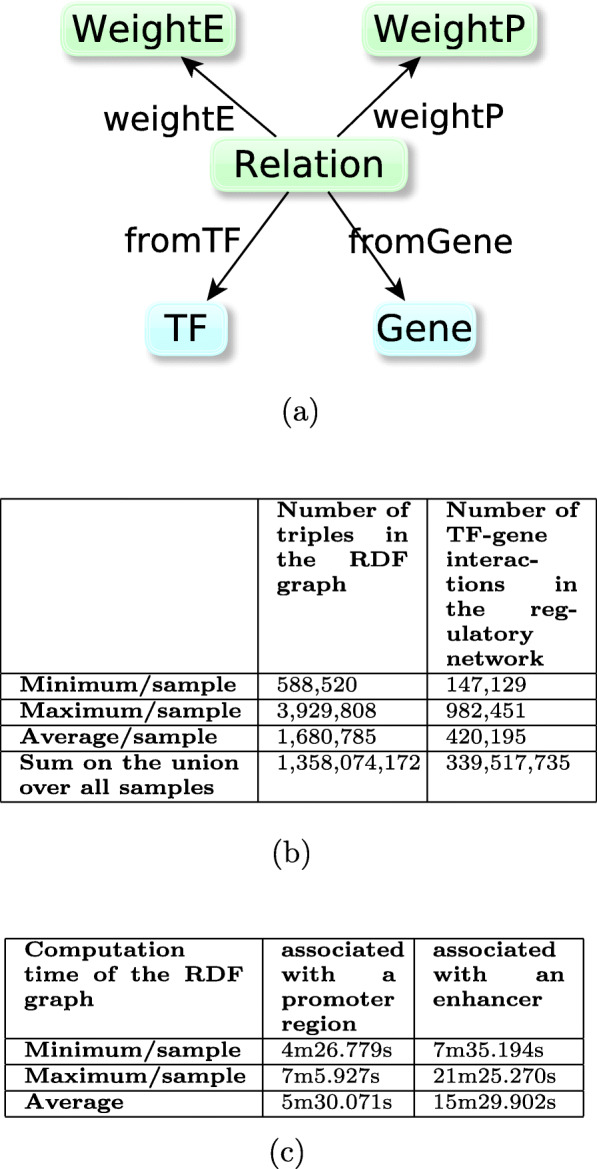
**a** RDF-model for each sample-specific graph: boxes represent classes of entities; a relation between a TF and a gene is characterized by (i) WeightP, the score which models the ability of the regulation to be operated through a promoter region, and (ii) WeightE, the score which models the ability of the regulation to be operated through an enhancer region. At least one of these scores has a nonzero value. **b** Description of the content of the 808 sample TF-gene regulatory networks. Each network is represented by an RDF graph based on the RDF model described in (**a**). The number of TF-gene interactions in the corresponding regulatory network is the number of instances of the “Relation” class in the graph. **c** Execution times of the SPARQL queries Insert_Enhancer.rq and Insert_Promoter.rq (cf. Fig. 2 and github repository) for contructing the graphs in (**b**), calculated for the first 102 samples. Structure of the 808 sample-specific graphs representing the parameters of the TF-gene regulation relations

The RDF model for sample-specific TF-gene graph is shown in Fig. 4, together with the characteristics of the RDF graphs, their associated TF-gene interaction networks, and their computation times. The 808 computed samples-specific graphs each contain between 147,129 and 982,451 relations between TF and genes, with an average of 420,195 relations in a given sample-network. Each relation is weighted with a promoter weight and an enhancer weight. This resulted in 808 RDF graphs composed of 1.7M triples in average - between 588K and 3.9M in a given graph. In total, the 808 sample-specific graphs contain 888,602,040 triples. Even if these sample-specific networks were considered as intermediary (and unpublished) results in the original *Regulatory Circuits* pipeline, we advocate that they are crucial for computing tissue-specific networks and therefore need to be accessible.

### Computation and addition of 394 weighted TF-gene interactions tissue-specific graphs

As described in the Regulatory Circuits project, each tissue is associated with 1 to 33 samples. It is therefore relevant to build TF-gene regulatory networks at the tissue scale by aggregating the information provided at the sample scale. A tissue-specific graph is the result of a SPARQL query with a UNION pattern on the source biological data (to identify TF and genes entities) and all the sample-specific networks (among the 808 described in the previous paragraph) associated with the considered tissue. As in sample-specific networks, TF-gene relations are first characterised by (i) a promoter weight (class *WeightP*) and (ii) an enhancer weight (class *WeightE*). They are obtained as the maxima of the corresponding weights of the same relation in the RDF graphs specific for all the samples constituting the tissue.

**Fig. 5:**
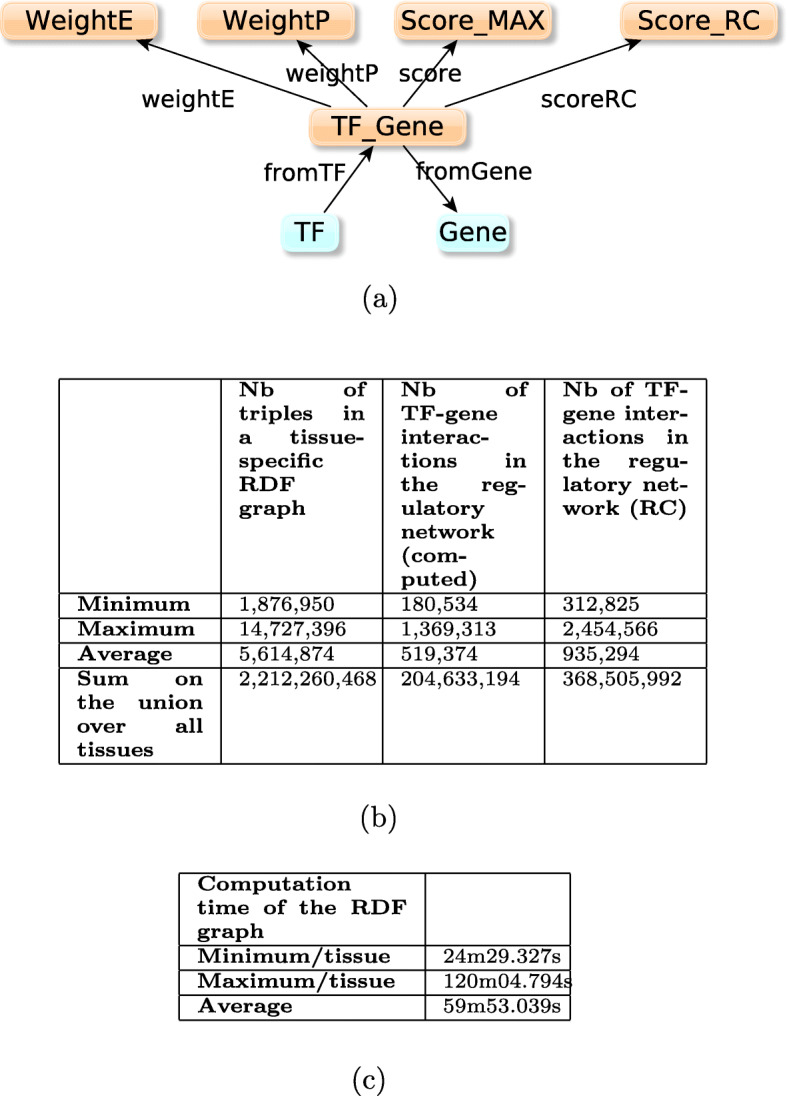
**a** RDF model for each tissue-specific graph: boxes represent classes of entities; a relation between a TF and a gene is characterized by (i) weightP, the score which models the ability of the regulation to be operated through a promoter region, (ii) weightE, the score which models the ability of the regulation to be operated through an enhancer region, (iii) a score composed of the maximum of weightE and weightP, and (iv) the score of the relation given by *Regulatory Circuits*. At least one of these scores has a non-zero value. **b** Description of the content of the 394 tissue TF-gene regulatory networks. Each network is represented by an RDF graph based on the RDF model described in (**a**). The number of TF-gene interactions in the corresponding regulatory network is the number of instances of the “Relation” class in the graph. **c** Execution times of the queries insert_tissue X.rq where X depends on the number of samples in the tissue (cf. Fig. 3 and github repository) for contructing the graphs in (**b**), calculated for the first 55 tissues networks. Structure of the 394 tissue-specific graphs representing the parameters of the TF-gene regulation relations

For the sake of further studies and to follow the original *Regulatory Circuits* networks, these two weights (weightP & weightE) may need to be combined in a single score. In this direction, the *Regulatory Circuits* project provided a global score for each TF-gene interaction in a tissue, which was integrated in our resource as the *RC score*. Unfortunately, no information is available on the formula used to compute this score. In order to gain in flexibility, we introduced the possibility to enrich the resource with additional aggregating scores: for instance, we computed the maximum of the two weights *weightP* and *weightE*, integrated in the resource as the *Max Score* (see Fig. 5 for the model of the RDF graph).

Based on the approach described in Methods section, we computed the RDF graph of each 394 tissue-specific TF-interaction network. As shown in Fig. 5, the RDF graphs’ sizes were heterogeneous (from 1,8 million triples to 14,7 Millions triples with an average of 5,6 million triples). They could be computed in 59 minutes in average. Together, the triples of each individual tissue-specific graph led to a resource of 2,212,260,468 triples.

From the RDF graph, we considered that a TF-gene interaction was present in the tissue if the score aggregating promoter and enhancer weights was strictly positive. According to the scores extracted from *Regulatory Circuits*, each tissue interaction network has between 312,825 and 2,454,566 TF-genes interactions. This is twice larger than the number of interactions in the networks obtained according to the *Max score* (between 180,534 and 1,369,313) and we have no explanation for the TF-genes interactions that have a positive score according to *Regulatory Circuits*, even though the corresponding scores in all the associated samples are zero.

All the TF-genes interactions, with their weights and scores, computed or from the original networks are stored in their respective tissue-specific named graphs and are available for querying following the SPARQL patterns available on GitHub.

### Modular organization of all the resources associated with regulatory circuits: the *LERC* dataset

**Fig. 6:**
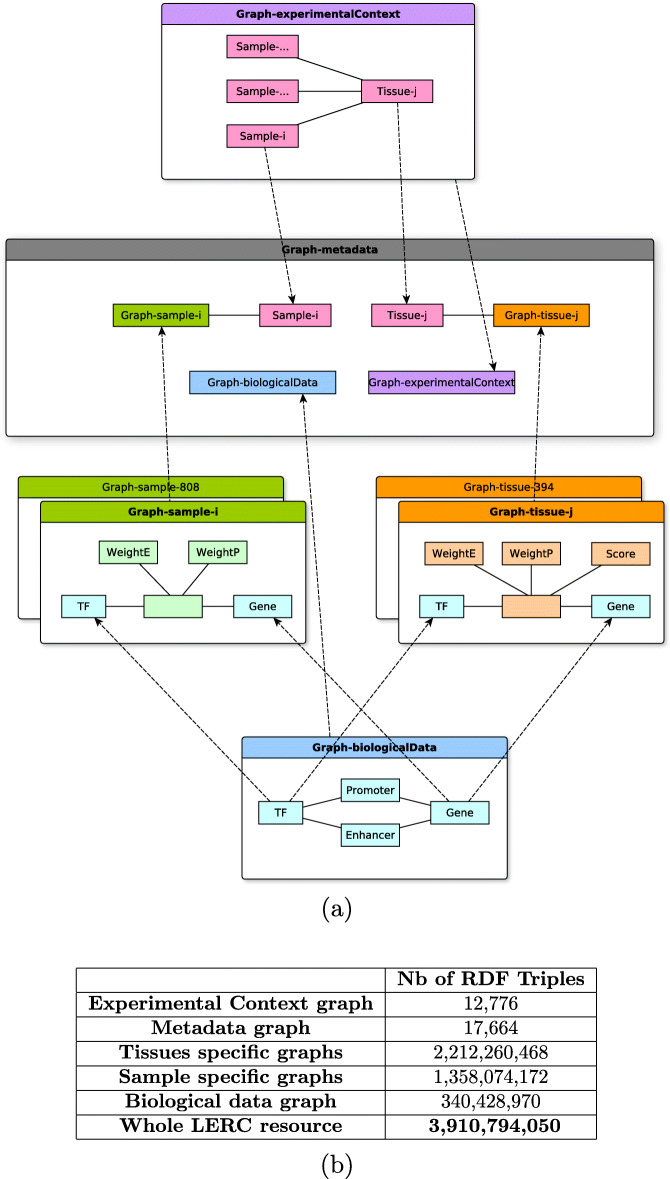
**a** The named graphs are the labelled boxes. Within them, the plain boxes and edges represent a simplified view of the main classes of each graph and their relations. The dotted edges represent how named graphs are linked: they relate identical URIs of entities in two different graphs. **b** Population in number of triples of the different graphs constituting the LERC resource. Number of triples for sample-specific and tissue-specific graphs given as the union of all the graphs of the given category. Modular organization of the RDF dataset into 1,205 named graphs

As seen previously, the initial RC dataset [14] can be associated simultaneously to (i) an RDF experimental context graph (see Methods section), (ii) 808 sample-specific RDF graphs, and (iii) 394 tissue-specific RDF graphs. In order to allow for a transversal exploration and to avoid performance issues when integrating and querying large datasets, we integrated all these resources in a single RDF dataset. This dataset is organized according to a modular architecture based on named graphs and shown in Fig. 6. The different layers of the modular organization are linked by an RDF *metadata graph*, which contains all the information about the other graphs including their VoID descriptions and characteristics about the graphs (number of triples, entities, etc.), as well as the associations of the samples and tissues with their respective graph. The RDF model is supported by the regulatorycircuits.owl ontology provided on the GitHub repository (cf. Availability section).

**Fig. 7:**
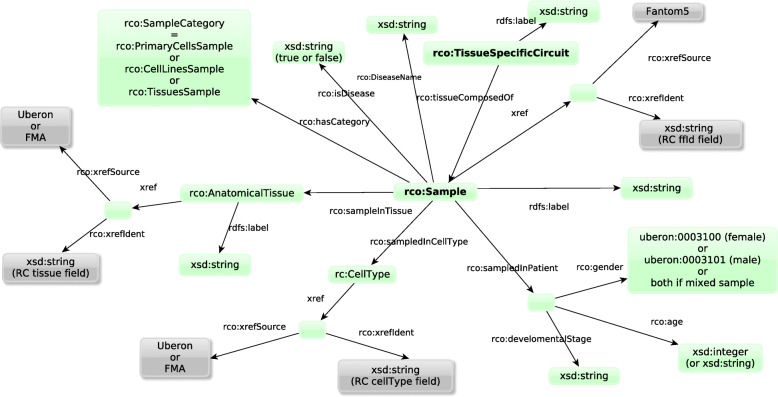
Structure of the experimental context graph describing the samples and the tissue-specific circuits (in bold). Green boxes represent classes of entities. Grey boxes represent mappings to external resources
- Each *sample-specific graph* provides the weights of the TF-gene interactions associated with the considered sample (See Fig. 4).
- Each *tissue-specific graph* provides the weights and scores of the TF-gene interactions associated with the considered tissue-specific regulatory networks, which is an aggregation of biologically related individual samples (See Fig. 5).
- The *metadata graph* contains all the information about the other graphs including their VoID descriptions, number of triples and entities.

Overall, the *LERC* dataset encompasses a total of 1,205 graphs of five types: 1 source biological data graph (in blue), 1 experimental context graph (in purple), 808 sample-specific graphs (in green), 394 tissue-specific graphs (in orange) and 1 metadata graph (in grey). Their main characteristics are as follows:

- The source *biological data graph* representing the biological data of the *Regulatory Circuits* and FANTOM5 projects was already published in [14] (see Fig. 1).
- The *experimental context graph* contains all the information about samples and tissues. As shown in Fig. 7, it describes the experimental information about the 808 samples (cell types, organs, patient, diseases...) and mappings to reference databases such as Uberon) and the 394 tissues (links to the samples they are composed of).

The *LERC* resource is composed of 3,910,794,050 triples and the distribution of the triples by graphs can be seen in Fig. 6. In total it required 28.6 days CPU on a 32GB RAM virtual machine with 4 VCPU to generate the RDF dataset from the initial integration of the biological data graph to the computation of the tissue-specific graphs. Overall, 118 anatomical tissues and 55 cell types were automatically mapped to 165 anatomical structures from the FMA and 157 from Uberon. 112 regulatory circuits were manually mapped to 79 diseases from the Human Disease ontology.

### Biologically-relevant queries

Exploring *LERC* can be done at several levels. We introduce fifteen examples SPARQL queries that can be used to navigate *LERC*. These pre-built queries are available on the GitHub of the project. They can be used as such or adapted to compose more elaborate queries.

- On the biological data graph, the query find_tf.rq allows to retrieve the different entities: TFs, genes or regions.
- Using the experimental context graph, the query tissues_samples.rq allows to extract the samples corresponding to a tissue or to all the tissues.
- The query extract_network.rq extracts all the TF-genes relations of a tissue (or sample) and its associated scores or weights, using its associated RDF graph.
- Using two different sample-specific graphs (or tissue), the query compute_network_2samples.rq enables to extract the union of the scored relations TF-genes for two samples (or tissues).
- On a sample or tissue named graph, the query find_targets.rq enables to find the targets of a specific TF in this sample (or tissue).
- Using two sample or tissue-specific RDF graphs, the query compare_targets.rq compares the targeted genes of a specific TF across two different tissues. It gives the union of the TF-genes relations and allows to compare their scores in the two tissues.
- The query compare_regulator.rq finds the different weights of the common regulators of a set of genes in two given tissues using two named graphs (tissue or sample specific).
- The query get_tissues_info.rq retrieves the samples composing a tissue, the biological tissue, age of the patients and other experimental information, using the experimental context graph.
- The query Limit_exp_conditions.rq limits the experimental conditions by retrieving the names of the samples composing a tissue that follow some restrictions: for example selecting only the samples from patients who are over 55 years old.
- The query Limit_to_healthCondition.rq lists all the samples composing one biological tissue with a disease or an health condition (non specific) associated and the name of the condition.
- The query Limit_to_healthy_samples.rq lists all the samples composing one tissue that are not linked to any disease.
- The query Samples_specific_disease.rq lists all the samples that are linked to a specific disease or diseases containing one word.
- The queries query-samplesInAnatomicalLocation.rq, query-samplesInCellType.rq and query-samplesInDisease.rq are federated queries that leverage the mappings to Uberon, the Cell Ontology and to the Human Disease ontology to retrieve the samples that match criteria requiring some ontology-based reasoning.

**Fig. 8:**
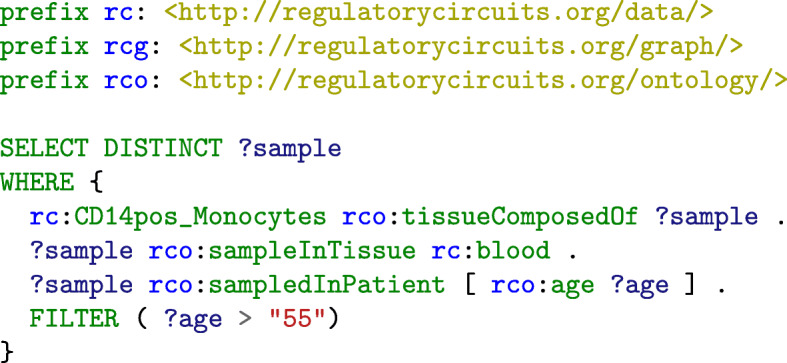
SPARQL query for retrieving the set of samples that meet some conditions expressed by the user (here, identify the subset of the “CD14+ Monocytes” samples taken in the blood of patients over 55 years old)

The Limit_exp_conditions.rq query illustrates the added-value of *LERC* in terms of flexibility to tailor analyses according to the needs of the user. For example, let us consider the tissue-specific TF-gene interaction graph “CD14+ Monocytes” which contains 2,402,498 scored TF-genes relations in *LERC*. However, we notice that the tissue is composed of 33 samples. Among these samples, only 3 were measured in blood (for the others, the biological origin is unknown), in men aged 47, 57 and 53 (result of the query get_tissues_info.rq). If the user is specifically interested in CD14+ Monocytes from blood for patient over 55, the experimental context graph can be queried with the query Limit_exp_conditions.rq. The query returns a unique sample respecting the conditions. The user can then use the query extract_network.rq with this sample. The resulting TF-gene interaction graph contains 846,930 interactions, pointing out the putative specific effect of genes on this tissue and patient category. We notice that such a tailored result is obtained with a very short SPARQL query (Fig. 8) which can be easily adapted to the user’s needs.

If the user wants to add to *LERC* the result of this new network computed with specific conditions, it can be done by using one of the injection queries (see Methods section and Fig. 2) replacing the samples’ names by the ones respecting the conditions and naming the resulting graph with a new specific name. Naturally, this should be performed on the user’s local copy of the dataset.

For the sake of further studies and to follow the original *Regulatory Circuits* networks, these two weights (weightP & weightE) may need to be combined in a single score.

## Discussion

In this article, we address the issue of making the biological datasets from the *Regulatory Circuits* project [6] reusable. We exposed the intermediate results such as the sample-specific regulation networks so that biologists can now access the information they need. This adds both the capacity to reuse portions of the pipeline’s results in other studies, and the capacity to enrich the data with additional information.

According to the Findability, Accessibility, Interoperability, and Reuse (FAIR) guidelines [18], the construction of the *LERC* resource to represent and extend the *Regulatory Circuits* project as an RDF dataset follows the best practices, using reification for weighted relations, and using named graphs to link the RDF graphs created for each different dataset handled in the regulatory circuits project. It also provides mappings to reference databases such as UniProtKB, Ensembl, Uberon, the Foundational Model of Anatomy ontology (FMA) and the Human Disease Ontology and follows the faldo chromosomal localization format. Federated SPARQL queries can then be used to combine information for *Regulatory Circuits* with information from these resources (e.g. associations with diseases, cell types or anatomical location. Examples illustrating federated queries and ontology-based reasoning are provided in the Github respository). The *LERC* resource is available on a persistent domain and all queries are publicly available on GitHub.

By both converting and enriching the *Regulatory Circuits* dataset into RDF with our modular principles, and by allowing flexible SPARQL queries on the *LERC* resource, our contribution to reusability is threefold. First, it *facilitates the reuse of Regulatory Circuits results in other studies* by providing access to the tissue-specific regulatory networks and the associated information. Second, it *facilitates the reuse of the studies’ data in other pipelines* by providing access to the samples’ experimental context and to the intermediary results such as the sample-specific regulatory networks, which can be reused to compute other indicators than *Regulatory Circuits* published weights and scores. Third, it *provides the capacity to enrich the Regulatory Circuits dataset with additional information* as the data model and Semantic Web technologies support adding new samples or defining new tissues, and the SPARQL queries we provide can generate the corresponding weights and scores. Overall, the *Regulatory Circuits* case study confirms that Semantic Web technologies are a relevant solution for reusing knowledge bases [12, 19], and demonstrates that they are also applicable to address the challenge of integrating them to project-specific datasets [20].

**Improving the exploration of*Regulatory Circuits*’s biological data and networks** In a previous work [14] we showed that the *Regulatory Circuits* workflow can be described using Semantic Web technologies thus increasing its reproducibility. This new implementation, including not only the input data but also the TF-gene interaction networks resulting from the in-silico integration of source biological data, allows a more flexible (re)use of *Regulatory Circuits*. The implementation we propose allows a fine-grained exploration: the user can select portions of the network, for example excluding regions at a lower distance than the *Regulatory Circuits* threshold, or excluding one type of region (e.g. for taking into account that promoters relations are more reliable).

**Improving the reusability and the enrichment of the source biological data with SPARQL queries** The networks available in the *Regulatory Circuits* website are static and cannot be updated when the biological datasets it was based upon evolve. A major advantage of our approach is that TF-gene interaction networks for samples and tissues are generated with SPARQL queries from the source biological data before being inserted in the resource. In addition, this implementation also allows the user to easily change some parts of the pipeline that generates the network, such as new calculation of the ranks, to add new genes or transcription factors in the networks, or to remove some of them. All these changes can easily be implemented by adapting the few available queries used to generate the network but necessitate to re-compute the new networks.

Our resource and the approach we use to populate it also facilitates the generation of new TF-gene interaction networks for new tissues, through the aggregation of samples with characteristics different from those chosen in the *Regulatory Circuits* project. *Regulatory Circuits* presents 394 tissue-specific networks, but looking into the detail of the samples and tissues revealed that some tissues could be separated into smaller sets. For example, the “CD14+ Monocytes” network given in *Regulatory Circuits* is based on 33 CD14+ monocytes cell samples, which have different characteristics (origin, donor age...). The modular structure of our resource allows for the computation of new TF-gene interaction network using these characteristics to better discriminate the samples.

Finally, using the *LERC* introduced in this article and the strategy used to build it allows the user to add new tissues or TFs if they have similar input data. This would require to pre-compute rankings for transcripts and regulatory regions which are at the moment provided by the *Regulatory Circuits* resource and cannot be recomputed. Similarly, introducing a new TFs would require to introduce new confidence values for their binding to regulatory regions.

**Improving interoperability** Among the 217 articles citing *Regulatory Circuits*, at least 42 either use directly the resulting networks for biological data explanation or use them as comparison for regulatory network inference. In 10 of these, *Regulatory Circuits* was used in combination with one or several other databases. Other resources on TF-genes relations exist [21, 22] but are complementary of *Regulatory Circuits*, the latter being the only one categorizing tissue-specific networks. By representing *Regulatory Circuits* as an RDF graph we therefore improve its interoperability with resources of similar scope already based on Semantic Web technologies, which facilitates its reuse in combination with other already existing RDF resources. In particular, it significantly extends the portion of FANTOM5 data available as RDF [23, 24].

## Conclusion

We present a generic approach for the enrichment of source biological data with the result of data analyses. Our results show that a Semantic Web approach scales not only for the integration of large-scale biological data but also for the iterative enrichment of such a resource with the results of in-silico analyses modeled with SPARQL queries. This is possible by using a modular structure based on RDF named graphs. This strategy could be easily transposed to other large scale life science studies which analysis pipeline describes relations with simple arithmetic functions.

## Data Availability

Original datasets of the Regulatory Circuits project, see [5]. *Linked Extended Regulatory Circuits (LERC)* resource, see https://regulatorycircuits-lod.genouest.org. Set of predefined SPARQL queries, mappings and ontology, see https://github.com/mlouarn/RCsparql/. LERC Turtle files, see [25].

## Abbreviations

FAIR: Findable Accessible Interoperable Re-Usable
FMA: Foundational Model of Anatomy
TF: Transcription factor
TFBS: Transcription factor binding site
LERC: Linked Extended Regulatory Circuits
